# Enhanced Bioaccessibility of Microencapsulated Puerarin Delivered by Pickering Emulsions Stabilized with OSA-Modified Hydrolyzed *Pueraria montana* Starch: In Vitro Release, Storage Stability, and Physicochemical Properties

**DOI:** 10.3390/foods11223591

**Published:** 2022-11-11

**Authors:** Zafarullah Muhammad, Rabia Ramzan, Ruifen Zhang, Dong Zhao, Nazia Khalid, Mei Deng, Lihong Dong, Mahwash Aziz, Rizwana Batool, Mingwei Zhang

**Affiliations:** 1 Sericultural & Agri-Food Research Institute, Guangdong Academy of Agricultural Sciences, Key Laboratory of Functional Foods, Ministry of Agriculture and Rural Affairs/Guangdong Key Laboratory of Agricultural Products Processing, Guangzhou 510610, China; 2 Department of Food Science and Technology, Government College Women University, Faisalabad 38000, Pakistan; 3 College of Food Science and Technology, Huazhong Agricultural University, Wuhan 430070, China

**Keywords:** *Pueraria montana* starch, OSA modification, microencapsulation, puerarin, Pickering emulsions, in vitro release

## Abstract

Puerarin is a bioactive flavonoid isolated from Kudzu roots that possesses numerous health benefits. However, its poor bioavailability and existing complex delivery systems with safety issues are challenging tasks for its incorporation into functional foods. Preparing modified-starch-stabilized Pickering emulsions containing microencapsulated puerarin with improved bioaccessibility was the key objective of the present research work. Acid-hydrolyzed high-amylose *Pueraria montana* starch (PMS) was modified with octenyl succinic anhydride (OSA) and evaluated as an emulsifier to prepare emulsions. The FTIR, SEM, and XRD results showed that PMS was successfully modified. Furthermore, the emulsification index (EI), mean droplet size, and ζ-potential values showed that modified starch with a higher degree of substitution (DS) enhanced the storage stability of emulsions. Similarly, the retention degree and encapsulation efficiency results of puerarin proved the assumption after storage of 16 d. The Pickering emulsions also helped in the controlled release of microencapsulated puerarin in vitro. The study outcomes proved that Pickering emulsions stabilized with OSA-modified PMS have promising applicability in functional foods as efficient food-grade delivery systems, enhancing oral supplementation and accessibility of puerarin.

## 1. Introduction

Puerarin is the phytoestrogen and main biologically active isoflavone chiefly present in the roots of Pueraria lobata [1], which is a Chinese leguminous edible and medicinal herb, traditionally known as Gegen or East Asian arrowroot in China [2]. According to the Chinese Pharmacopoeia, puerarin is presented as (8-(β-D-Glucopyranosyl)-4′,7-dihydroxyisoflavone), and its content should not be less than 13% in P. lobata. Puerarin is cool in nature, with a sweet, pungent smell and thirst-quenching properties, making it a suitable isoflavone to be used for functional foods [3,4]. According to several modern studies, puerarin has the potential effects of decreasing blood pressure, blood sugar, and blood lipids [2,5]. Due to these pharmacological properties, puerarin exerts antiapoptotic, antiosteoporosis, anti-inflammatory [6], antioxidant, hepatoprotective [7], anticancer, antidiabetic, antiobesity, and anti-myocardial ischemic effects [8]. Numerous studies on cell lines and animal models indicated that puerarin could impact different cardiovascular diseases, such as cardiac hypertrophy, myocardial infarction, atherosclerosis, hypertension, and ischemic stroke [8,9]. The mechanisms primarily involve suppression of apoptosis, inflammation, and oxidative stress while fostering endothelial, neurological, and cardiac functions [10,11]. Therefore, the capabilities of puerarin make it a potential neuroprotective and cardioprotective isoflavone in nature [8,12].

Due to all these potential benefits, puerarin possesses great interest in being incorporated into functional foods as a health-improving nutraceutical [13]. According to some studies, the recommended safe dietary dose of puerarin for humans is 200 to 400 mg/day [14]. Due to its chemical structure, puerarin is a hydrophobic isoflavone. It has poor solubility in water (1.1 × 10^−2^ mol/L) and gastrointestinal fluids, making its oral bioavailability approximately 7%, which is very low for harvesting its beneficial effects [15,16]. The maximum solubility of puerarin in phosphate buffer at pH 7.4 is 7.56 mg/mL, and its aqueous solubility is 0.46 mg/mL [15,17,18].

Furthermore, according to some studies, orally administered puerarin with a dose of 10mg/kg has a shorter half-life of approximately 0.80–1.00 h, a lower partition coefficient of 1.95, and a Cmax value of 140–230 μg/L in vivo [17,19]. Owing to these characteristics, its permeability in epithelial cells of the small intestine is very low and prone to oxidation reactions when exposed to enzymes, acids, and thermal environments, resulting in lower bioavailability and serum levels [20]. Mainly, puerarin is administered through intravenous injections, and it is widely distributed in mammary glands, liver, kidneys, spleen, and stomach [21]. Because of its poor solubility and elimination half-life, most of its quantity is excreted through urine, so multiple and high doses of injections are needed. Solubility of puerarin can be improved by using cosolvents, such as polyvinylpyrrolidone and propylene glycol [22,23]. However, frequent high doses of intravenous injections and cosolvents have controversial safety issues and adverse reactions, such as allergy, fever, and erythrolysis. All these discrepancies have made pressing concerns about its commercial values, oral bioavailability, and limited applications in nutraceutical and functional food industries [3]. Summing up the mentioned points, the development of an oral delivery system with stable microencapsulation of puerarin will significantly resolve several issues. According to the latest available information, only a few studies are available for the microencapsulation of puerarin, such as nanocapsules, nanoparticles, and protein hydrogels. These reasons are interest-provoking for us to develop a modified-starch-based novel delivery system for puerarin microencapsulation.

In recent years, the formulations for oral administration with improved absorption of isoflavones have attracted widespread acceptability and adaptability. Finding a novel efficient formulation and a safe administration method for puerarin delivery is critical. This challenging task could be overcome by microencapsulating the nutraceuticals within food colloids. This technique provides protection to unstable bioactive compounds and increases their bioavailability by retaining them within a microencapsulating membrane or matrix [24]. Emulsification is among the commonly used microencapsulating techniques in which two immiscible phases are mixed. These mixtures are thermodynamically unstable because the dispersed droplets coalesce, resulting in destabilization through phase separation. The interface between emulsion droplets needs surface active or amphiphilic compounds, biopolymers, or surfactants for stabilization. These stabilized emulsion droplets can potentially be used to microencapsulate the unstable bioactive compounds, enhancing their applicability in various food, pharmaceutical, and cosmetic products [25,26].

Particle-stabilized or Pickering emulsions have gained significant scientific and industrial attention due to their higher stability against destabilization mechanisms (Ostwald ripening and coalescence) [27]. The coalescence is arrested due to the formation of a thick barrier at the interfaces of the large-sized droplets. Second, Ostwald ripening is arrested due to the formation of interfacial pressure and capillary forces at closely packed adjacent particles, which, in turn, impede mass transfer across the interface. In addition, Pickering emulsions (PE) have higher biocompatibility than surfactant-stabilized emulsions because side effects, such as toxicity and anaphylaxis, could be avoided by using PE [28,29,30]. As an alternative and promising approach, it could be considered that puerarin could be dissolved in MCT (medium-chain triglyceride) to fabricate oil-in-water Pickering emulsions. This approach is popular for protecting and administering food-grade hydrophobic bioactive substances as dietary supplements.

These emulsions are stabilized by a variety of particles, such as modified starches, protein nanogels, kaolinite, nanocellulose, and kafirin. Starches are natural polymers and have extensive utilization in Pickering emulsions as emulsifiers, with several advantages, such as low cost, FDA approval, toxicity, bioavailability, biocompatibility, mucoadhesiveness, gastro resistance, pH responsiveness, and degradability by colonic microbiota [31]. Naturally, the starches are hydrophilic, whereas these should have partial affinity with both oil and water phases. Therefore, these need to be modified for optimized emulsification efficiency. For this reason, starch esterification with dicarboxylic acids to generate octenyl succinic anhydride (OSA) esters is found to be one of the most effective approaches [32]. Due to its hydro- and lipophilicities, OSA-modified starch has been found to be an excellent emulsifying substance to microencapsulate hydrophobic bioactives, such as lutein [33], resveratrol [34], β-carotene [35], and curcumin [36]. Generally, high-amylose corn, potato, cassava, wheat, quinoa, and several other modified starches have been used for the purpose. These starches have different properties to stabilize the Pickering emulsions based on different inherent characteristics, such as molecular weight, size, degree of branching, and crystal helix structure [37,38]. In several studies, OSA-modified high-amylose corn starch was studied to assess the stabilization of O/W Pickering emulsions, enhancement in oral bioavailability, and facilitate intestinal epithelial transport of alpha-lipoic acid (ALA) [39,40,41]. However, OSA-modified stabilized Pickering emulsions containing microencapsulated puerarin with potential bioaccessibility have rarely been studied. Based on these investigations, *Pueraria montana* starch as a non-conventional substance has been studied for OSA modification and production of highly stabilized O/W Pickering emulsions.

Additionally, acid hydrolysis has also been used as a pretreatment of starch before OSA modification to obtain unique properties for better stabilization of the emulsions [42,43]. Acid hydrolysis advantageously generates more functionalizing points for attachment of ester groups by changing the surface charge of the starch and increasing its hydrophilicity without destroying the granule structure [41,44]. Acid hydrolysis only changes the physicochemical characteristics of the starch and potentially influences the gelatinization temperature, which is another reason to incorporate this pretreatment in the present study. 

Keeping in view the above background, the study proposes the development of OSA-modified-starch-based Pickering emulsions for microencapsulation of puerarin as a dietary supplement for oral administration. These emulsions could be utilized in functional foods for better biological activities by enhancing the bioavailability of puerarin and compensating for its deficiency in daily intake. Moreover, the effects of acid hydrolysis on the DS and emulsifying characteristics of OSA esterification with different concentrations on hydrolyzed *Pueraria montana* starch were studied. Additionally, the physicochemical characteristics of microencapsulated puerarin in Pickering emulsions, including loading capacity, microencapsulation efficiency, in vitro release, pH, and storage stabilities, were investigated. 

## 2. Materials and Methods

### 2.1. Materials

*Pueraria montana* starch was procured from the Key Laboratory Functional Foods, Guangdong Academy of Agricultural Sciences, Guangzhou. MCT oil was obtained from Jarrow Formulas, Los Angeles (CA 90035-4317), USA. Octenyl succinic anhydride with 97% purity was purchased from Sigma Aldrich, Co., 3050 Spruce Street, St. Louis, MO, USA, whereas puerarin with a purity of ≥97% was procured from the Aladdin industrial corporation, Shanghai, China. MilliQ water was used to prepare solutions and emulsions. All other chemicals were of analytical grade.

### 2.2. Modification and Characterization of Pueraria montana Starch

#### 2.2.1. Pretreatment of PMS with Acid Hydrolysis

Acid hydrolysis of the PMS was carried out before OSA modification. The previously described method in [45] was used with slight modifications for the pretreatment. Briefly, *Pueraria montana* starch (10 g, dry weight basis) was pretreated with 1.0 M HCl in a 250 mL beaker. Incubation of the mixture was carried out in a water bath at 50 °C for 2, 4, 6, 8, 12, and 24 h. The mixtures were stirred with magnetic stirrers after specific intervals. Then, 5% NaOH was used to neutralize the incubated samples. Afterward, the samples were centrifuged at 4000× *g* for 5 min and washed with MilliQ water to obtain a neutral pH. Then, absolute ethanol was used twice to wash the samples by centrifuging for 5 min at 4000× *g*. Finally, the hydrolyzed starch samples were freeze-dried for 72 h. The dried starch precipitates were ground finely to pass through a 100-mesh sieve. The sieved starch powders were put into screw-capped tubes and kept in a desiccator for further use.

#### 2.2.2. OSA Modification of PMS

After acid hydrolysis pretreatment, the *Pueraria montana* starch was subjected to OSA modification using the method described in [46]. Aqueous solutions (35% *w*/*w*) of PMS were prepared using MilliQ water. The solutions were continuously mixed with a magnetic stirrer. Then, using the 0.5 M NaOH solution, the pH of the starch slurries was adjusted to 8.0 and maintained at 8.0 ± 0.2 during the whole reaction time. Afterward, the OSA with contents of 2%, 4%, 6%, and 8% of starch was diluted five times with absolute ethanol. The diluted OSA was mixed drop by drop for 2 h in the previously prepared starch mixtures. The process was performed at 35 °C for 3 h. After finishing the modification procedure, the pH of starch mixtures was adjusted to 6.5 using 0.5 M HCl. Then, washing of the suspension was completed twice using MilliQ water and twice with 70% ethanol by centrifuging at 3300 rpm for 30 min. After centrifugation, the precipitates were freeze-dried for 73 h, grounded, sieved through 100-mesh size, labeled, and stored for further analyses. 

### 2.3. Determination of DS 

The average number of OH groups substituted per glucose unit is termed the degree of substitution (DS). A slightly modified titration method in [47] was used to determine the DS of OSA-modified PMS. In short, OSA-modified PMS (5 g) was suspended in 0.1 M HCl (50 mL) and continuously stirred for 30 min. Afterward, centrifugation of the suspensions was completed for 30 min at 3300 rpm, and first washing was completed with 90% ethanol solution and then twice with MilliQ water. Finally, the suspensions of starch and water (300 mL) were prepared at boiling temperature for 20 min with continuous stirring in a water bath and then titrated with 0.1 M NaOH. The indicator was phenolphthalein, and native PMS was taken as a reference. Equation (1) [46] was used to determine the percentage of carboxyl groups from OSA-modified PMS.

(1) $$\%OSA=\frac{\left(V_{sample}-V_{blank}\right)\times M\times 210}{W}\times 100$$

*V_sample_* is the NaOH volume (L) used for sample titration, whereas *V_blank_* is the NaOH volume (L) used for blank titration. M represents the molarity of NaOH, *W* is the weight (g) of starch, and 210 denotes the molecular weight of the octenyl succinate group.

Then, Equation (2) [46] was used to determine the DS

(2) $$DS=\frac{162\times \%OSA}{210\times 100-\left[\left(210-1\right)\times \%OSA\right]}$$

### 2.4. Particle Size Determination of Modified PMS

Modified and native starch samples were analyzed for particle size using a laser particle size analyzer (WJL-628, INESA, Shanghai, China).

### 2.5. X-ray Diffraction (XRD) Patterns of Starches

The characterization of OSA-modified PMS and native starch powders for XRD patterns was completed by using the X’Pert³ Powder diffractometer (Malvern Panalytical company, Cambridge, UK). The range of diffraction scattering angles (2θ) was 5°–60°. Measurement of the relative crystallinity was conducted using the software of Jade 6.0 (Jade software corporation, York, UK).

### 2.6. FTIR Spectroscopy

FTIR spectroscopic analysis was carried out using the method of [40] with the help of Bruker Vertex 76 FTIR spectrophotometer (Bruker Optik GmbH., Ettlingen, Germany) to characterize the functional groups of native and modified PM starches with different OSA concentrations. The instrument was equipped with a KBr beam splitter and detector (mercury cadmium telluride) using a Platinum Diamond ATR accessory coupled with diamond crystal with a 45° angle of incidence. The spectra were collected at a wavenumber region of 4000–500 cm^−1^ using 32 scans at a resolution of 4 cm^−1^. The spectra were analyzed using Thermo Scientific OMNIC FTIR software (Thermo Scientific corporation, Shanghai, China).

### 2.7. Morphological Analysis Using SEM (SEM)

The morphological features of the modified and native starch samples were studied by using the ZEISS Merlin Field Emission Scanning Electron Microscope. The starch samples were fixed with carbon tape on aluminum stubs, gold plated, and 10 µm images were taken under 1.00 Kx resolution and 5 kV accelerated voltage.

### 2.8. Preparation of Pickering Emulsions Stabilized by Modified Pueraria montana Starches

#### 2.8.1. Formulation of Pickering Emulsions

OSA-modified PMS samples were used as specific emulsifiers for preparation of O/W Pickering emulsions. Further, 5% modified starch samples were gelatinized at 90 °C for 20 min to prepare a continuous phase, whereas 5 mg/L puerarin was mixed in MCT (10%) and used as a dispersed phase. First of all, puerarin-loaded coarse emulsions were prepared using a rotor-stator (IKA, T25 digital ultra turrax, Guangzhou, China) at 10,000 rpm for 5 min. The MCT and puerarin mixture were poured drop by drop during homogenization. Then, these coarse emulsions were homogenized in a high-pressure homogenize machine (ATS Homogenizer, AH-BASIC, Shanghai, China), completing 3 cycles at 150 MPa.

#### 2.8.2. Determination of the Emulsification Index

The emulsions were stored for 16 d at 4 °C, and the preparation day was taken as the first day of storage. The emulsification index of the emulsions was recorded on 1, 8, and 16 d by using the method as described by Zheng et al. [48], following Equation (3).

(3) $$\mathrm{EI}=\frac{Height\;of\;the\;emulsified\;layer}{Total\;height\;of\;the\;emulsion\;mixture}$$

#### 2.8.3. Droplet Size Distribution and Confocal Laser Scanning Microscopy

The distributions of droplet size of Pickering emulsions were determined on days 0, 8, and 16 using a laser size analyzer (Baxter BJ-9300HJ, Dandong Baxter Instrument Inc., Dandong, China). The droplet size distribution was determined immediately after preparing the emulsions. Then, 3 mL of each emulsion was put into 5 mL centrifuge tubes and stored at room temperature. During the storage period, droplet size distributions (DSDs) of emulsions were measured again to assess their stability (the samples for analyses were taken from the middle of the tubes). The results of the DSDs were also used to calculate the median droplet diameter values (the size of emulsion droplets bigger than that of half the population and smaller than that of the other half is referred to as the median diameter) [49]. Additionally, the microstructures of the selected emulsions were determined by the previously described method [49] using a confocal laser scanning microscope (Leica SP8, Leica microsystems Inc., Heidelberg, Germany). Nile red (10 mL) was used to dye the crude emulsions (100 mL) for 30 min while they were in the dark. The oil droplets were then examined under the microscope.

#### 2.8.4. Measurement of ζ-Potential

Measurement of the ζ-potential of emulsions was determined by a Zetasizer Nano Series (Zetasizer Nano-ZS90, Malvern Instruments, Worcestershire, UK). After diluting the samples with MilliQ water, the emulsions’ pH (3.0, 6.0, and 9.0) was set using HCl and NaOH. Then, the samples were filled into capillary cells to measure zeta potential. The samples were diluted with deionized water and filled into the test cell before measurement. This experiment was performed to check the stability of emulsions on different pH as well. Each sample took three readings with triplicate measurements.

#### 2.8.5. Retention Degree and Microencapsulation Efficiency (MEE)

The retention degree of the emulsions was determined after 16 d of storage, whereas the microencapsulation efficiency of puerarin was determined after the freshly prepared emulsions. The dissolved puerarin in MCT was taken as control (puerarin–MCT) for the comparison of retention degree after 16 d. Puerarin was extracted using the OSA couple liquid–liquid extraction method. In short, we took 2 mL ethyl acetate in a 5 mL centrifuge tube and added 500 μL emulsions, then sonicated for 20 min and centrifuged for 5 min at 8000 rpm. Then, the residue of (500 μL) supernate was dried with a nitrogen gas stream, and the dried residues were redissolved into acetonitrile. Then, the filtration of the samples was conducted using a 0.45 μm membrane for HPLC analysis. Retention degree and MEE were determined with the following equations.

(4) $$Retention\;degree\;\left(\%\right)=\frac{C_{10d}}{C_{actual}}\times 100$$

(5) $$MEE\;\left(\%\right)=\frac{C_{actual}}{C_{added}}\times 100$$

In these equations, *C_actual_* is the amount of puerarin loaded in the lipid droplets of the emulsions, whereas *C_added_* is the amount of total puerarin used during the preparation process, and *C_10d_* is the amount of puerarin retained in the emulsion after storage of 16 d.

#### 2.8.6. Measurement of In Vitro Release Profile of Puerarin

The dialysis bag diffusion method was used to determine the in vitro release profile measurements of puerarin from the emulsion samples by following the slightly modified method [50]. Concisely, the emulsion samples (5 mL) were taken in dialysis bags and immersed in 50 mL PBS (pH 7.4) containing ethanol (10% *v*/*v*) with continuous magnetic stirring (200 rpm) at 37 °C, and 1 mL of aliquots was taken out and replaced with an equal volume of freshly prepared PBS at specific time intervals of 2, 4, 6, 8,10, 20, 30, 40, and 50 h. Extraction of the released puerarin from the emulsions was conducted and analyzed with HPLC.

#### 2.8.7. HPLC Measurement

An Agilent HPLC system (1260 series) equipped with Hypersil GOLD-C18 column (250 × 4.6 mm, 5 μm particle size, Thermo Fisher Scientific Co., Ltd., Bellefonte, PA, USA) was used to perform the quantitative analysis of puerarin by following the method as described in [51]. The pH of the mobile phase was set at 7.0, and it consisted of 0.1% acetic acid in the water and 0.1% acetic acid in acetonitrile (90:10, *v*/*v*), and the mobile phase was degassed before running the column, maintaining the temperature at 25 °C. The run time was set for 20 min, with an injection volume of 20 µL, a flow rate of 1 mL/min, and a wavelength of 254 nm.

### 2.9. Statistical Analyses

The data were compiled by completing entire analyses in triplicate. Statistics 8.1 (Analytical Software, New York, NY, USA) and the Tukey method were used for comparison of means and ANOVA (analysis of variance). The difference was taken as significant statistically when *p* < 0.05. Values of means and standard errors were considered and shown in charts as coordinate pairs with error bars.

## 3. Results and Discussion

### 3.1. Acid Hydrolysis Pretreatment and Degree of Substitution of Modified Starches

Esterification of the starch was carried out with 2% OSA after acid hydrolysis pretreatment for 2, 4, 6, 8, 12, and 24 h (Table 1). The results for the degree of substitution (DS) are shown in Table 2. The results shown in Table 1 reveal that the DS was increased with increased hydrolysis time. The highest DS value (0.96 ± 0.06) was observed after a 24 h pretreatment. It was demonstrated that the subsequent esterification reaction benefited from depolymerization of starch. Therefore, a 24 h acid hydrolysis condition for the esterification of starch was used in this study.

Different OSA concentrations (2, 4, 6, and 8%) were used to modify the high-amylose *Pueraria montana* (Kudzu) starch after acid hydrolysis. The results for DS are shown in Table 2. With increasing concentrations of the OSA, the results for DS were also increased: 0.67 ± 0.02–1.80 ± 0.03. These results were in line with the previously reported range of OSA degree of substitution (3–9%) [52,53]. There is a greater boost in DS with a higher concentration of OSA because the chances of molecular collisions between the reactant molecules are increased as well. Meanwhile, after increasing the OSA concentration from 9 to 12%, the DS becomes smooth. This might happen due to the production of steric inhibition upon introduction of the OSA groups, which hinders the OSA accessibility to the starch molecules [53,54].

### 3.2. X-ray Diffraction Analysis

The diverse polymorphic forms of starch categorized as A, B, and C types are based on the semi-crystalline structure and X-ray patterns of the starch granules. According to [55], the primary association of starch crystallinity is with the amylose content (which has the main distribution in the amorphous region) and the arrangement of amylopectin double helices.

**Figure 1:**
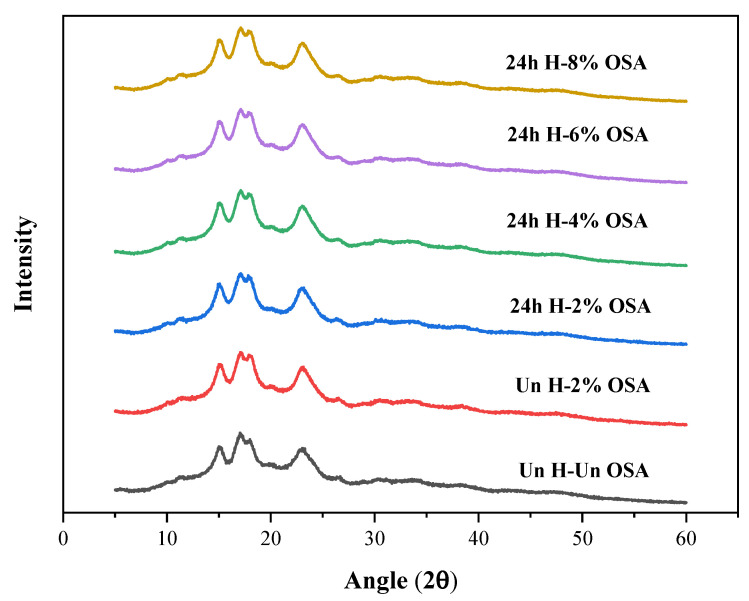
XRD patterns of OSA-modified and native high-amylose PMS (Kudzu) starch samples.

XRD patterns of the native and acid-hydrolyzed high-amylose PMS (Kudzu) starches are presented in Figure 1. PMS has the B-type crystalline structure as its XRD patterns show corresponding diffraction peaks (2θ) at 15°, 22.9°, and a doublet at 17.1° and 17.92° (Figure 1). A similar pattern was shown by the Un-3% OSA starch, demonstrating that the esterification of OSA took place without crystallinity changes, mainly in the amorphous regions of starch granules [40,56]. However, following the pretreatment with acid hydrolysis, the diffraction peak at about 2θ grew weaker in comparison to other modified starches, which might indicate the presence of amylose. A possible explanation might be the reduction in amylose content due to the destruction of the amylose structure by the acid hydrolysis pretreatment [54]. By breaking down amylose, acid hydrolysis decreases the overall crystallinity, decreases the molecular weight of starch, and increases the amount of free aldehyde groups [57].

### 3.3. Scanning Electron Microscopic (SEM) Analysis

**Figure 2:**
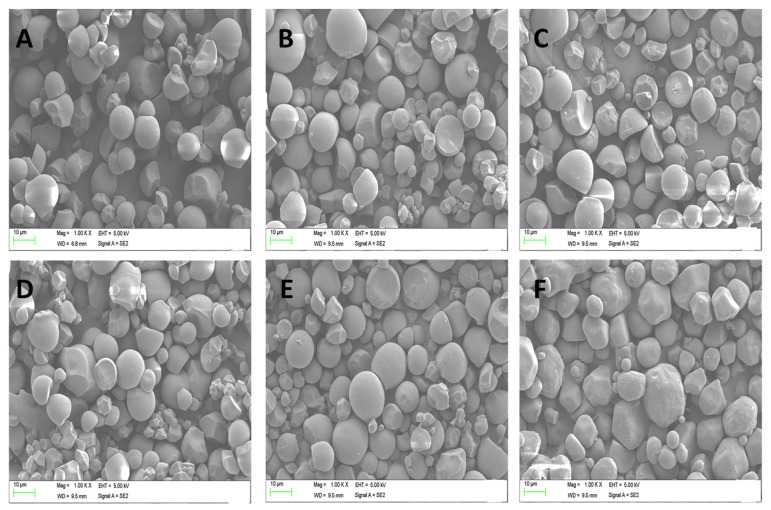
Scanning electron micrographs of OSA-modified high-amylose PMS (Kudzu) starch samples, (**A**) native, (**B**) Un-2% OSA, (**C**) 24 h H-2% OSA starch, (**D**) 24 h H-4% OSA starch, (**E**) 24 h H-6% OSA starch, and (**F**) 24 h H-8% OSA starch.

The SEM morphological analysis of the native and modified starch granules can be viewed in Figure 2. According to Figure 2A, native PMS had a smoother surface and irregular polyhedral shape. However, the acid-hydrolysis-pretreated and modified starches had extra-small starch fragments, and their surfaces were somewhat roughened and cracked (Figure 2B–F). Meanwhile, Figure 2B shows the uneven granular size and irregular structure of the PMS. It was due to the aggregation and destruction of the native starch during the processes of debranching and gelatinization [58,59]. The OSA-modified PMS starch granules showed partially aggregated and roughened structures compared to the native starch due to the increased degree of substitution (Figure 2C,F) [60]. This effect was attributed to the fact that, due to the addition of the OSA to the dispersion, the pH of the reaction system was decreased, and esterification introduced bifunctional hydrophilic and hydrophobic groups, which would lead to granule fusion and rough surface of the starch. Furthermore, an obvious reduction in the size of the starch granules was observed. This phenomenon was analogous to the study conducted in [61]. The fact that some of the starch granules were severely harmed and, at the same time, some appeared to be unharmed suggested that there was a heterogenous esterification reaction. 

### 3.4. FT-IR Analysis

**Figure 3:**
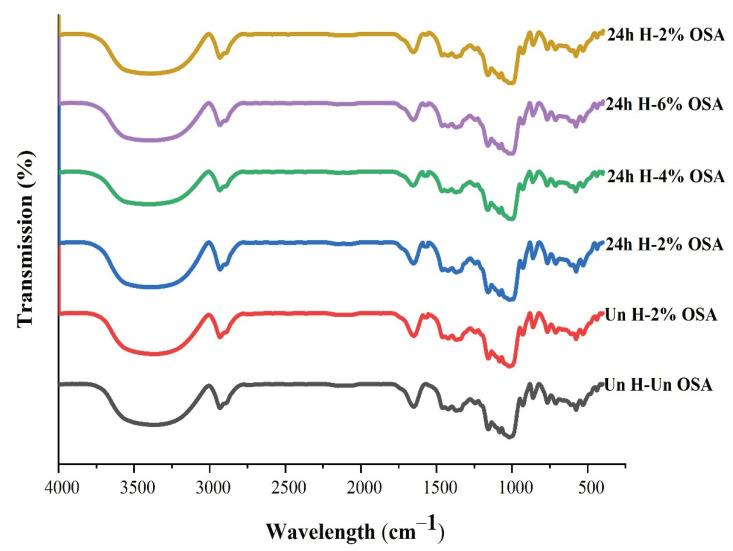
The FTIR diffraction pattern of OSA-modified high-amylose and native PMS (Kudzu) starch samples.

The impact of esterification with various OSA additions on the structure of PMS starch was investigated using infrared spectra analysis. The spectra between 500 and 4000 cm^−1^ were studied, as shown in Figure 3. With increasing additions of OSA relative to DE, the intensity of the band at 3488 cm^−1^, which was attributable to the O-H stretching vibration absorption, decreased. The intensity of the band associated with the O-H stretching vibration absorption decreased with increased additions of OSA at 3488 cm^−1^ compared to DE. This might be due to the esterification of the O-H band to the carbonyl group. The antisymmetric stretching of C-H in the -CH_2_ and CH_3_ groups caused the absorption peak at about 3000 cm^−1^. Additionally, new distinguishing peaks were given to the spectra of the OSA-modified starch as compared to the native starch at around 1609 cm^−1^ and 1565 cm^−1^ [62,63,64]. The respective asymmetric stretching vibrations of the ester carbonyl and carboxylate RCOO− caused these changes, illustrating that the ester carbonyl and carboxyl groups replaced hydroxyl groups in the native starch. Similar results were shown in other studies [65,66], which demonstrated that increased OSA concentration for modification did increase the intensity of these bands.

### 3.5. Distribution of Particle Size

**Figure 4:**
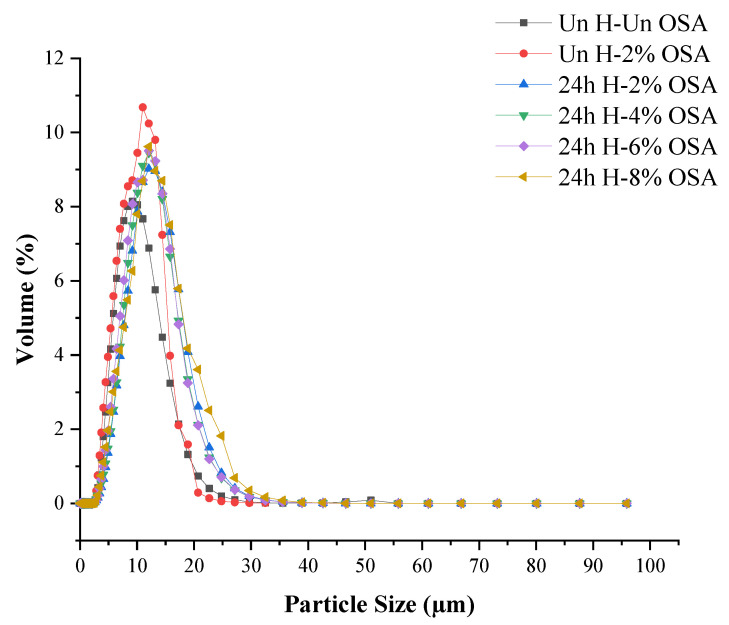
Mean granule size of native and OSA-modified high-amylose PMS (Kudzu) starch samples.

The median granular size (D50) of high-amylose PMS was 8.14 μm in the beginning, but it was decreased after the pretreatment and OSA modification, as shown in Figure 4. The decrease in median size is beneficial as it increases the emulsifying capacity of the OSA-modified starches and also reduces the droplet size. Generally, acid hydrolysis and OSA pretreatments are recommended in combination to increase the yield of starch particles. On the basis of these results, it can be speculated that stabilized emulsions can be produced by using the OSA-modified starch particles [67,68]. Smaller starch particles were typically produced by acid hydrolysis and OSA modification, which could then result in smaller emulsion droplets. The size decrease of the starch particles led to a rise in the emulsion index for the acid-hydrolyzed starch. The size of the droplets and the creaming of Pickering emulsions appeared to be somewhat affected by the shape of starch [69].

### 3.6. Stability of Emulsions

The prepared Pickering emulsions were stored for 1, 8, and 16 days, and their emulsification index (EI) values are given in Table 3. The higher EI values were shown by the acid-pretreated OSA starches, whereas the lowest EI values were shown by the Un-2% OSA starch during the evaluation period. The acid hydrolysis pretreatment increased the generation of functionalizing sites for the OSA groups on starch molecules. These functionalizing points were attracted to the oil phase and allowed the starch granules to create a dense membrane that prevented the coalescence of the oil droplets [70]. The oil droplets of various modified starches migrated to the surface when the EI values decreased over time due to the density differences between the two phases and the settling of starch granules, and it affected the state of emulsification [70]. It was observed that, as the OSA addition was increased from 2 to 8% *w*/*w*, the emulsifying index of the hydrolyzed and OSA-modified starches was also increased. The increasing degree of substitution (DS) also increased the oleophilic groups, which resulted in an improvement in the emulsion index. The improved EI of OSA-modified and hydrolyzed starches with higher DS resulted in easier adsorbance to the oil–water interfaces. Due to this phenomenon, higher steric hindrance and denser interface film have occurred between the emulsion droplets [53,71].

**Figure 5:**
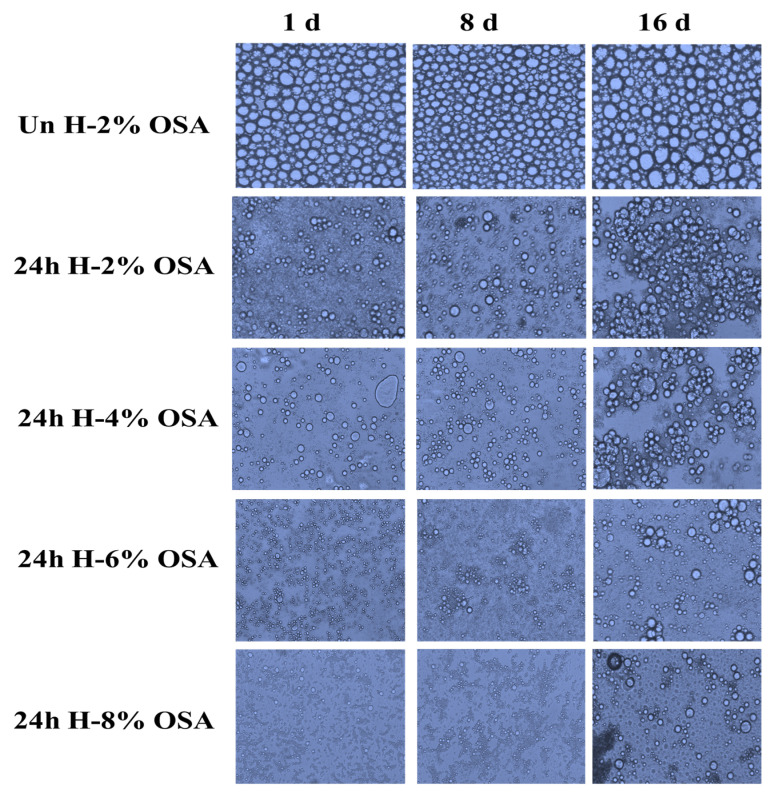
Microstructures of emulsions stabilized by OSA-modified Kudzu starch stored for 1, 8, and 16 d.

**Figure 6:**
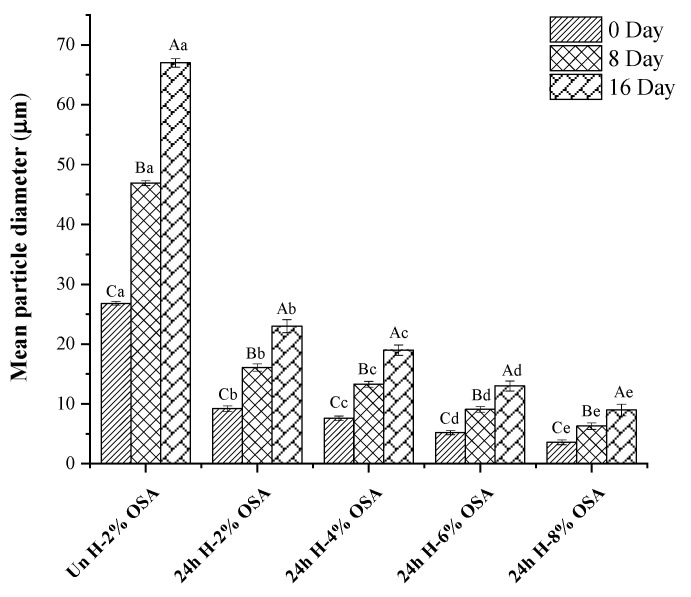
Mean droplet size of Pickering emulsions stabilized with OSA-modified PMS starches for the storage time of 0, 8, and 16 d. Samples shown with different capital letters (A, B, C) indicate a significant difference (*p* < 0.05) when the same samples were compared for different storage times. Samples indicated with different lower-case letters (a, b, c, d, e) showed a significant difference (*p* < 0.05) when the different samples were compared for the same storage time.

The state of the emulsion depicted in Figure 5 and Figure 6, respectively, can also be reflected by droplet size distribution and emulsion microstructures in addition to EI values. In comparison to other emulsions on days 1, 8, and 16, the emulsion stabilized by Un-2% OSA starch had the highest mean droplet sizes (26.8, 46.9, and 67 m), demonstrating an insufficient granule–oil interaction, which was unable to stabilize the emulsions successfully. These results were in line with the EI values. The smallest mean droplet size was evidently produced by the 24 h H-8% OSA emulsion. It may be attributed to higher emulsion stability since a larger DS indicated the presence of more lipophilic groups, which allowed the adsorption of starch granules on the oil–water interface. The oil droplets were confined in the dense and multi-layered starch particles, which reduced their attraction to one another and increased the steric hindrance [53,54]. In the meantime, as time passed, the average droplet size increased, as shown in Figure 5. It happened because more hydrophobic OSA starch granules were accumulated in the aqueous dispersion [70]. The fact that they were able to easily build a dense layer over the droplets to stop coalescence after the OSA modification could also be used to explain this phenomenon. The size of the droplets generated during homogenization and the droplets’ resistance against aggregation and flocculation were the key determinants of the particle size of the emulsions. The emulsion particle size was reduced by breaking up oil particles under increased pressure as a result of OSA-modified starch’s ability to lower the interfacial tension between oil and water. In the meantime, OSA dextrin was simpler to create a thick barrier at the oil–water interface to fend off emulsion flocculation and aggregation than starch or unmodified dextrin [53,59]. According to a study by Pan et al. (2019) [53], curcumin was more effectively encapsulated in dextrin-stabilized emulsions (57.93%) than it was in native starch-stabilized emulsions (11.88%). With an increase of 6% to 12% concentration of OSA, there was an increase of 72.51% to 85.41% in the microencapsulation efficiency of curcumin observed in the OSA-dextrin-stabilized emulsions [72,73].

### 3.7. ζ-Potential of Emulsions

**Figure 7:**
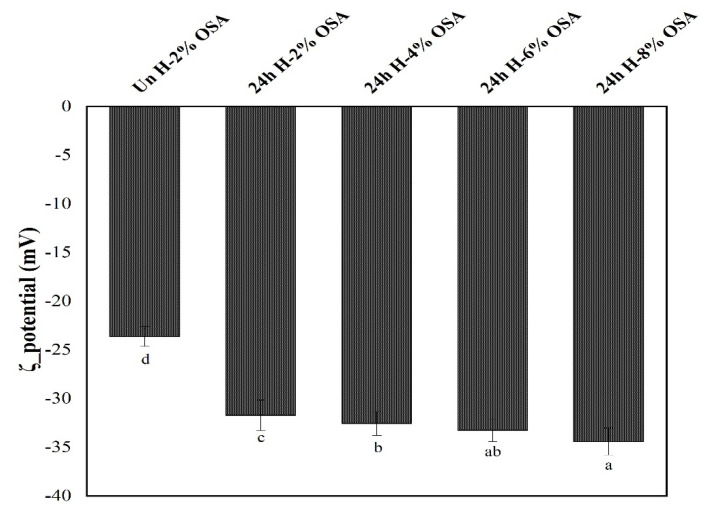
ζ-potential of fresh emulsions stabilized by high-amylose PMS (Kudzu) starch with different OSA concentrations. The significant difference of the same indicator is indicated with different letters in different samples (*p* < 0.05).

Zeta potential, which is used to assess how strongly particles repel or attract one another, is a key criterion for evaluating the dispersion stability of colloids [53]. Figure 7 presents the results of the ζ-potential of fresh emulsions. The lowest absolute value of ζ-potential (23.62 ± 1.02 mV) was observed in the emulsion made with Un H-2% OSA starch, whereas the highest value (34.40 ± 1.39 mV) was shown in the emulsion prepared with 24 h H-8% OSA starch. In a continuous medium, the higher the absolute value of ζ-potential, the higher will be the free (Brownian) mobility of the dispersed phase, preventing the formation of aggregates in the dispersed phase [40]. Principally, with increased concentrations of the OSA, the absolute values of the ζ-potential were also enhanced. The outcomes were in line with [53], pointing to greater charge density on the modified starch surface.

**Figure 8:**
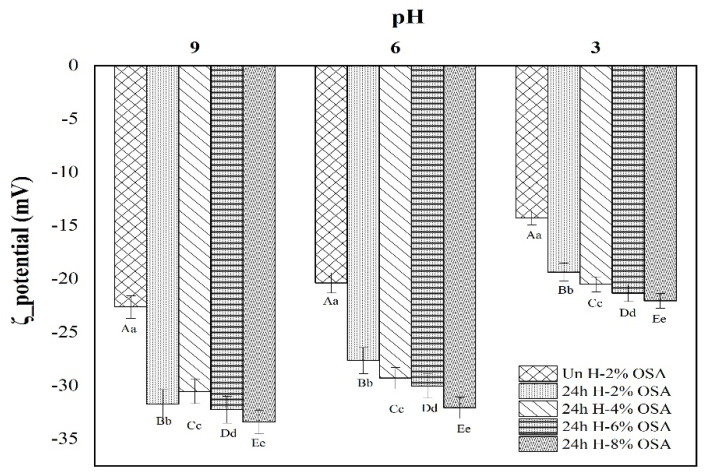
ζ-potential of the five emulsions at pH 4.0, 6.0, and 8.0. A significant difference (*p* < 0.05) is indicated in different samples designated with different capital letters (A, B, C, D) while comparing for the same pH. Meanwhile, a significant difference (*p* < 0.05) is shown in samples designated with a different lower-case letter (a, b, c) while comparing between same samples at different pH.

Additionally, the five emulsions were also investigated for their stability behavior at a pH range of 3, 6, and 9, and Figure 8 indicates the results. The ζ-potential absolute values rose as pH rose, and the emulsions at pH 9.0 showed greater ζ-potential absolute values. This trend showed that emulsions are more stable at a higher pH. Similarly, emulsions prepared with OSA-modified starch with a higher DS were more stable. Overall, the steric barrier between the starch particles and the weak polyelectrolyte caused by the carboxyl groups could be credited with the stability of emulsions [70]. As reported previously, the ζ-potential absolute values were lower. The droplets might be inclined to aggregation when the pH was below 6.0 because electrostatic repulsion between the droplets was insufficient to overcome the attractive force needed to keep the emulsions stable. Additionally, the stability profile is also enhanced with a weak polyelectrolyte caused by carboxyl groups. The stability of acid-hydrolyzed OSA-stabilized Pickering emulsions can also be affected by a negative charge [46,54]. Hence, steric hindrance and electrostatic repulsion might be the mechanisms for acid-hydrolyzed OSA starch to produce stabilized Pickering emulsions, whereas an appropriate higher pH could be advantageous. Altogether, Un H-2% OSA-based emulsions were highly unstable. However, emulsions prepared with OSA-modified PMS have stability in the following order: 24 h H-2% OSA < 24 h H-4% OSA < 24 h H-6% < 24 h H-8% OSA.

### 3.8. Retention Degree and Microencapsulation Efficiency (MEE) of Puerarin

**Figure 9:**
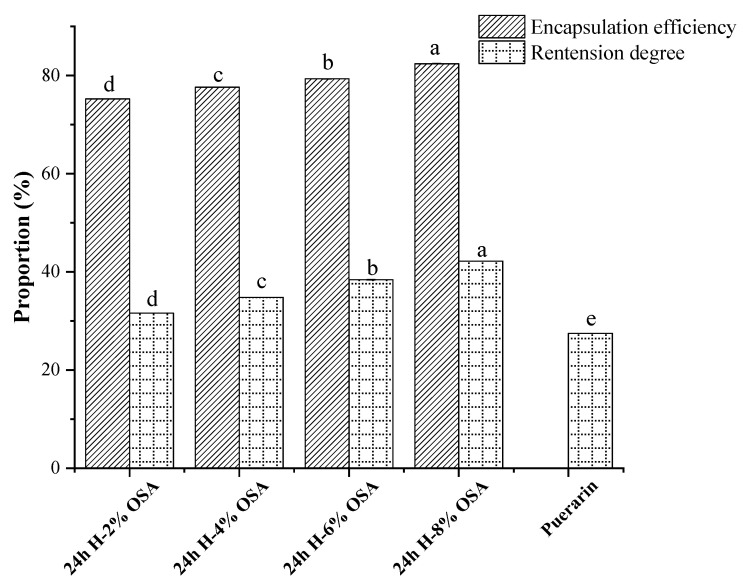
Retention degree and microencapsulation efficiency (MEE) of puerarin in samples before and after storage. A significant difference for the same indicator in different samples is indicated with different letters (*p* < 0.05).

As it was found that Un-2% OSA-starch-based emulsions were highly unstable, the puerarin was microencapsulated using other modified starches. Figure 9 shows the retention degree of fresh emulsions and microencapsulation efficiency of puerarin after 16 d storage. As shown in the results, the encapsulation efficiency of Pickering emulsions stabilized with OSA-modified (2, 4, 6, and 8%) PMS starch was high. The highest MEE (82%) for puerarin was shown by the 8% OSA-modified starch. The hydrophobic contact between the molecules may be improved by adding additional OSA groups, which would increase the loading of puerarin. A similar pattern was found in a study [53] where OSA-dextrin-stabilized emulsions showed MEE of 72.51% to 85.41% for 6 to 12% OSA concentrations, respectively. The retention degree of puerarin in 6% and 8% OSA-modified-starch-stabilized emulsions was 42% even after 16 d storage. Comparatively, the 2% and 4% OSA-starch-based emulsions had a very low content of puerarin, and the lowest was found in MCT.

The results showed that Pickering emulsions could be employed as an efficient delivery strategy for puerarin encapsulation since the emulsion stabilized by 8% OSA-modified starch with greater DS had superior stability. With an increasing OSA addition from 4 to 8% (*w*/*w*), the emulsifying stability of OSA-based starch emulsions was also increased. The increase in the oleophilic group with increased DS may be responsible for the improvement in emulsion stability, making it easier for higher-DS OSA starch to adsorb to the oil–water interface, resulting in a higher steric hindrance and a denser interface layer between the droplets in the emulsion [73]. 

### 3.9. In Vitro Release Profile of Puerarin

**Figure 10:**
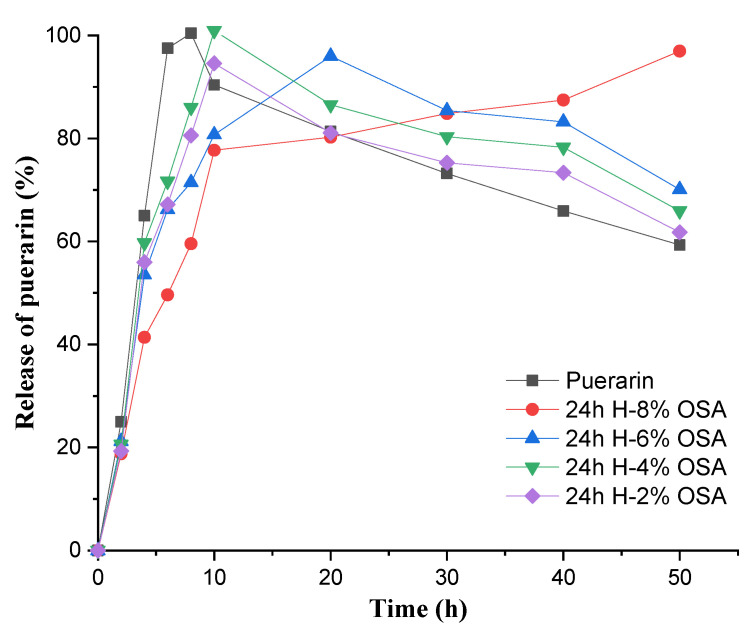
Profiles of in vitro release of Pue from different Pickering emulsions.

Puerarin has a shorter half-life in the body due to its poor solubility in water. Its sustained release effects and therapeutic efficacy can be increased by microencapsulation using biodegradable polymers. The drug release curves of puerarin microencapsulated with OSA-modified PMS are shown in Figure 10. It was discovered that there was a quick release of puerarin microparticles from the dialysis bags. Consequently, the isoflavone was completely dissolved in 6 h. Initially, there was a burst release (60.2%) of puerarin in the first 4 h after being microencapsulated by 24 h H-8 % OSA-modified PMS. These results reveal that there is easy removal of the puerarin fraction analogous to the encapsulation efficiency results. The in vitro release profiles of puerarin microencapsulated in several emulsions and PBS medium are shown in Figure 10. Evidently, there was a rapid and complete release of MCT-dissolved puerarin within 6 h, whereas, within 10 h, explosion effects of different degrees of puerarin occurred. After this, there was a gradual release of puerarin during the next 40 h, and a slightly downward releasing trend was observed by the 24 h H-2%, 4%, and 6% OSA-starch-stabilized emulsions. The complete release of about 96.02% at 50 h was revealed in 8% OSA-stabilized-emulsion only, whereas almost total release of puerarin was observed in other emulsions before 10 h. In vitro controlled release of puerarin was achieved in 24 h H-8% OSA-starch-stabilized emulsions. It is due to the higher DS of 8% OSA starch, which helps in efficient microencapsulation and stabilization of oil droplets in Pickering emulsions. This phenomenon allows these stabilized microcapsules to release puerarin in PBS medium continuously. Simultaneously, the amount of puerarin in the other three emulsions was subsequently lowered because the totally released puerarin in the medium was converted into its by-products as its releasing process was affected by the OSA concentration. A similar pattern was observed by the authors of [74], where the microparticles of puerarin encapsulated by poly(L-lactide) showed 89.65% release of puerarin within 24 h following a complete release in 36 h, maintaining a sustained release process.

## 4. Conclusions

OSA modification of high-amylose *Pueraria montana* (Kudzu) starch in combination with acid hydrolysis could make it an effective emulsifier to produce stabilized Pickering emulsions. Meanwhile, these emulsions could be successfully employed as a novel strategy to microencapsulate and deliver puerarin, which is a hydrophobic bioactive isoflavone. The details of the starch modification, preparation, and characterization of Pickering emulsions containing microencapsulated puerarin have thoroughly been provided in this research work. According to the findings, the stabilized Pickering emulsion with 24 h H-8% OSA starch showed better resilience against harsh conditions, superior storage stability, and microencapsulation efficiency (MEE) of puerarin. Furthermore, the controlled release of puerarin in vitro was successfully achieved in the emulsions containing 24 h H-8% OSA starch. The present research will be helpful in establishing the high-amylose PMS granules as alternative particle stabilizers to be incorporated in the stabilization of the Pickering emulsions. Additionally, this research will be helpful in expanding the applicability of puerarin and related bioactive compounds in the functional food industry by exploiting and fabricating novel ingredients to introduce new food-grade, economical, and orally administered delivery systems.

## Figures and Tables

**Table 1:** The effects of different time intervals of acid hydrolysis on the degree of substitution.

Samples	Hydrolysis Time (h)	OSA Content (%)	DS (%)
2 h H-2% OSA	2	2	0.70 ± 0.03 ^d^
4 h H-2% OSA	4	2	0.75 ± 0.02 ^c^
6 h H-2% OSA	6	2	0.81 ± 0.04 ^b^
8 h H-2% OSA	8	2	0.88 ± 0.05 ^b^
12 h H-2% OSA	12	2	0.91 ± 0.01 ^a^
24 h H-2% OSA	24	2	0.96 ± 0.06 ^a^

A significant difference of the same indicator is indicated with different letters in different samples (*p* < 0.05).

**Table 2:** The effects of different concentrations of OSA on the degree of substitution.

Samples	Hydrolysis Time (h)	OSA Content (%)	DS (%)
Un H-Un OSA	0	0	
Un-2% OSA	0	2	0.67 ± 0.02 ^d^
24 h H-2% OSA	24	2	0.96 ± 0.06 ^c^
24 h H-4% OSA	24	4	1.23 ± 0.04 ^b^
24 h H-6% OSA	24	6	1.64 ± 0.07 ^a^
24 h H-8% OSA	24	8	1.80 ± 0.03 ^a^

A significant difference of the same indicator is indicated with different letters in different samples (*p* < 0.05).

**Table 3:** The emulsification index (EI) of modified-PMS-starch-stabilized Pickering emulsions following different conditions on different days.

Samples	Emulsification Index		
	1 d	8 d	16 d
Un H-2% OSA	0.53 ± 0.02 ^d^	0.48 ± 0.01 ^d^	0.45 ± 0.00 ^d^
24 h H-2% OSA	0.92± 0.06 ^a^	0.91 ± 0.03 ^a^	0.90 ± 0.04 ^c^
24 h H-4% OSA	0.93 ± 0.03 ^a^	0.92 ± 0.04 ^a^	0.90 ± 0.02 ^b^
24 h H-6% OSA	0.93 ± 0.01 ^a^	0.92 ± 0.02 ^a^	0.91 ± 0.01 ^a^
24 h H-8% OSA	0.94 ± 0.05 ^a^	0.93 ± 0.04 ^a^	0.91 ± 0.02 ^a^

A significant difference of the same indicator is shown in different samples with different letters (compared with the same column, *p* < 0.05).

## Data Availability

Data is contained within the article.
